# Supplementary material for: Event-related theta and gamma band oscillatory dynamics during visuo-spatial sequence memory in younger and older adults
Source: PLoS One. 2024 Apr 2;19(4):e0297995. doi: 10.1371/journal.pone.0297995 (PMC10986947; doi:10.1371/journal.pone.0297995)
Supplement: Attachment — Submitted filename: PLOS1_R2_response to reviews.docx [file pone.0297995.s002.docx]

We appreciate the opportunity to submit this revised manuscript, “Event-related Theta and Gamma Band Oscillatory Dynamics During Visuo-spatial Sequence Memory in Younger and Older Adults” and thank the reviewers for their thoughtful comments. We respond to each of the reviewer’s enumerated points below. Our responses are indicated in bolded, italics font.

**Reviewer #1:**

No Comments

**Reviewer #2:**

1) One piece of new information provided, however, raises a new concern. Specifically in responses to my request "how many segments were excluded due to >10% bad channels" the authors respond "...Finally, segments with more than 7 (10%) channels marked as bad were excluded from the analysis, resulting in the removal of an average of one trial (SD = 2.96) per condition." My concern is that if the average was 1 but the SD was almost 3, then the distribution must be quite skewed across conditions? Is this accurate? And if so, does differential exclusion rates across conditions introduce a confound? All I'm asking for here is a little more detail (e.g., give the number per condition) and some comment on if/how it affects the interpretation of the data.

***First, we appreciate the reviewer’s concern with respect to the potential that varying the number of trials across conditions would introduce a potential confound. Fortunately, this concern is mitigated by the systematic selection of equal numbers of trials across all conditions for the analysis. This systematic selection is described at the bottom of page 17 in the original manuscript.***

***Second, we realized that reporting the variance across conditions is somewhat unusual and that reporting the descriptive statistics across subjects would be the more conventional way to report on the rejection of data. As a result, we have modified the description to include descriptive information over subjects and an analysis of variance to describe variability across conditions. Below is the revised description of data rejection that can be found in the middle of page 17 of the revised manuscript.***

***“Finally, segments with more than 7 (10%) channels marked as bad were excluded from the analysis, resulting in the removal of an average of 1.0 (SD = 2.2) trials per subject across all conditions. The number of trials rejected did vary significantly across the three encoding conditions, F(2,116) = 5.44, p < 0.01, but did not significantly differ across the three recall conditions, F(2, 116) = 2.23, p = 0.11. The potential confound introduced by varying the number of trials across sequence positions was mitigated by the systematic selection of an equal number of trials (described below).”***
